# Supplementary material for: Mental Health and Adherence to COVID-19 Protective Behaviors among Cancer Patients during the COVID-19 Pandemic: An International, Multinational Cross-Sectional Study
Source: Cancers (Basel). 2021 Dec 15;13(24):6294. doi: 10.3390/cancers13246294 (PMC8699048; doi:10.3390/cancers13246294)
Supplement: Supplementary file 1 [file cancers-13-06294-s001.zip › cancers-1439110-supplementary.pdf]

Supplementary Materials: Mental health and adherence to COVID-19 protective behaviors among cancer patients during the COVID-19 pandemic: An international, multinational cross-sectional study

Table S1. Health behavior data, COVID risk Self-Efficacy, Importance and Intentions, Coping (COPE B), Perceived Stress (PSS) data. Cognitive Affective, Mindfulness Scale data, Pro-Social Behaviour data, Brief Assessment of Family Functioning data, MSBS, Mental Health Continuum Short Form for Adults data, Positive and Negative Affect data, Illness (COVID) Perceptions data, Beliefs about COVID data, separately for cancer patients and free of cancer individuals.

		**Cancer participants**	**Non-cancer participants**	***p*-value**	**Effect size**
**Total Number of the Participants [*n*]**		264	9301		
**Quarantine/self-isolation Data**					
Weeks in quarantine or self-isolation		5 (4, 7)	6 (4, 7)	0.66[i]	0.08[k]
Since the quarantine began, have you needed to leave your house for work? [***n*** (%)][a]	No	136 (51.7)		0.37[j]	0.02[l]
	Once only	14 (5.3)	718 (7.7)		
	A couple of times	58 (22.1)	2209 (23.8)		
	More than 3 times per week	55 (20.8)	1951 (21.0)		
Have your finances changed since being on quarantine? [***n*** (%)][a]	Have got better	11 (4.2)	838 (9.0)	<01[j]	0.04[l]
	Stayed the same	176 (66.9)	5358 (57.6)		
	Have got worse	76 (28.9)	3104 (33.4)		
Have you been able to obtain all the basic supplies you have tried to buy (e.g., soap, food items)? [***n*** (%)][a]	Yes	248 (94.3)	8691 (93.5)	0.58[j]	0.01[l]
	No	15 (5.7)	609 (6.5)		
**Health Behavior**					
Do you smoke? [***n*** (%)][d]	Yes	32 (12.1)	1769 (19.0)	<01[j]	0.03[l]
	No	232 (87.9)	7531 (81.0)		

How many cigarettes did you smoke per day until the quarantine or self-isolation measures? [median (IQR)]		15 [8-20]	10 [5-15]	0.03[i]	0.38[k]
How many cigarettes did you smoke per day during the last week? [median (IQR)]		14.5 [10 0]	10 [4-20]	0.09[i]	0.33[k]
Difference of cigarettes before and current. [median (IQR)]		0 [-5- 0]	0 [0-4]	0.30[i]	0.00[k]
Do you consume alcohol? [N (%)][b]	Yes	110 (41.7)	4522 (48.6)	0.03[j]	0.02[k]
	No	154 (58.3)	4779 (51.4)		
How often did you consume alcohol per week until the quarantine or self-isolation measures? [median (IQR)]		2 [1-3]	2 [1-3]	0.04[i]	0.27[k]
How often did you consume alcohol per week before cups? [median (IQR)]		1 [1-1]	3 [1-4]	0.26[i]	0.63[k]
How often did you consume alcohol during the last week? [median (IQR)]		2 [1-4]	2 [1-3]	0.25[i]	0.22[k]
How often did you consume alcohol before cups? [median (IQR)]		1 [1-1]	3 [2-4.5]	0.60[i]	0.00[k]
Difference before and during. [median (IQR)]		0 [0-0]	0 [0-1]	0.52[i]	0.11[k]
Do you exercise? [N (%)][b]	Yes	175 (66.3)	6107 (65.7)	0.83[j]	0.00[l]
	No	89 (33.7)	3194 (34.3)		
How many times on average did you exercise per week until the quarantine or self-isolation measures? [N (%)][e]	Never	8 (4.6)	379 (6.2)	0.04[j]	0.04[l]
	Approximately 1-2 times	58 (33.1)	2579 (42.3)		
	Approximately 3-4 times	76 (43.4)	2264 (37.1)		
	At least 5-7 times	33 (18.9)	879 (14.4)		

How many times on average did you exercise during the last week? [N (%)][f]	Never	6 (3.4)	434 (7.1)	0.11[j]	0.04[l]
	Approximately 1-2 times	57 (32.6)	2208 (36.2)		
	Approximately 3-4 times	72 (41.1)	2119 (34.8)		
	At least 5-7 times	40 (22.9)	1333 (21.9)		
On average how long did you exercise during the last week? [N (%)][g]	Not at all	5 (2.9)	208 (3.4)	0.27[j]	0.03[l]
	15 minutes or less	14 (8.0)	583 (9.6)		
	15-30 minutes	41 (23.4)	1253 (20.6)		
	30-45 minutes	48 (27.4)	1278 (21.0)		
	45 minutes - 1 hour	33 (18.9)	1358 (22.3)		
	More than 1 hour	34 (19.4)	1401 (23.0)		
Exercise current before [median (IQR)]		0 [0-0]	0 [0-1]	0.92[i]	0.05[k]
How would you rate your sleep quality overall? [N (%)][h]	Very good	45 (17.2)	1412 (15.2)	0.39[j]	0.03[k]
	Fairly good	105 (40.1)	3764 (40.5)		
	Neither good not bad	55 (21.0)	2374 (25.6)		
	Fairly bad	48 (18.3)	1484 (16.0)		
	Very bad	9 (3.4)	249 (2.7)		
How much has your sleep changed since quarantine? [N (%)][h]	I sleep more	50 (19.1)	2941 (31.7)	<0.01[j]	0.02[l]
	I sleep less	57 (21.8)	2025 (21.8)		
	I sleep about the same	155 (59.1)	4317 (46.5)		
What do you think your total screen time is during the last week (include TV, Laptop, computer,		6 [4-9]	8 [5-10]	<.01[i]	0.29[k]

smartphone, tablet)? Please answer in hours per day. [median (IQR)]				
What do you think your total screen time before (include TV, Laptop, computer, smartphone, tablet)? [median (IQR)]	4 [2-8]	5 [3-8]	0.31[i]	0.17[k]
Difference before and current. [median (IQR)]	2 [0-4]	2 [0-4]	0.22[i]	0.10[k]
**COVID risk Self-Efficacy, Importance and Intentions**				
I have the skills to get through this difficult situation. [median (IQR)]	7 [6-7]	6 [6-7]	<.01[i]	0.17[k]
I can deal with this difficult situation. [median (IQR)]	7 [6-7]	6 [6-7]	0.02[i]	0.13[k]
When facing difficulties in following the recommendations, I am certain that I will overcome them. [median (IQR)]	7 [6-7]	6 [6-7]	<.01[i]	0.15[k]
Compared to other people, I can follow these recommendations pretty well. [median (IQR)]	7 [6-7]	6 [6-7]	<.01[i]	0.18[k]
Even when things get tough, I can follow these recommendations quite well. [median (IQR)]	7 [6-7]	6 [6-7]	<.01[i]	0.19[k]
COVID risk self-efficacy [median (IQR)]	6.6 [5.9-7]	6.2 [5.6-7]	<.01[i]	0.20[k]
How important do you think it is to follow the recommendations to participate in social distancing, or staying home as much as possible? [median (IQR)]	7 [6-7]	7 [6-7]	<.01[i]	0.08[k]
How much do you intend on following the recommendations for social distancing (stay at home	7 [6-7]	7 [6-7]	<.01[i]	0.16[k]

as much as possible) for the next week? [median (IQR)]				
**Coping (COPE B)**				
COPEB self-distraction [median (IQR)]	6 [4-7]	6 [4-7]	0.47[i]	0.02[k]
COPEB active coping [median (IQR)]	6 [5-7]	6 [5-7]	0.23[i]	0.13[k]
COPEB denial [median (IQR)]	2 [2-4]	2 [2-4]	1.0[i]	0.03[k]
COPEB substance use [median (IQR)]	2 [2-2]	2 [2-3]	0.35[i]	0.12[k]
COPEB use of emotional support [median (IQR)]	4 [3-6]	5 [4-6]	0.11[i]	0.10[k]
COPEB use of instrumental support [median (IQR)]	4 [3-5]	4 [3-6]	0.10[i]	0.15[k]
COPEB behavior aldis engagement [median (IQR)]	2 [2-3]	2 [2-4]	0.06[i]	0.14[k]
COPEB venting [median (IQR)]	5 [3-6]	5 [4-6]	0.73[i]	0.11[k]
COPEB positive framing [median (IQR)]	6 [4-7]	6 [5-7]	0.11[i]	0.05[k]
COPEB planning [median (IQR)]	6 [4.5-7]	6 [5-7]	0.28[i]	0.07[k]
COPEB humour [median (IQR)]	4 [3-6]	4 [3-6]	1.0[i]	0.03[k]
COPEB acceptance [median (IQR)]	7 [6-8]	7 [6-8]	<.01[i]	0.20[k]
COPEB religion [median (IQR)]	4 [2-6]	3 [2-5]	0.49[i]	0.11[k]
COPEB self-blame [median (IQR)]	3 [2-4]	3 [2-4]	0.44[i]	0.11[k]
**Illness (COVID) Perceptions data**				
How much COVID-19 does affects your life? [median (IQR)]	8 [6-9]	8 [6-9]	0.60[i]	0.06[k]
How long do you think COVID-19 will continue? [median (IQR)]	7 [6-8]	7 [5-8]	0.25[i]	0.17[k]
How much control do you feel you have over COVID-19? [median (IQR)]	7 [5-8]	7 [5-8]	0.73[i]	0.06[k]

How much do you think existing treatments help patients with COVID-19? [median (IQR)]	6 [4-7]	6 [5-8]	0.19[i]	0.17[k]
How much does COVID-19 worries you? [median (IQR)]	7 [5-9]	7 [5-8]	0.18[i]	0.11[k]
How well do you feel you understand what is happening with COVID-19 [median (IQR)]	8 [6-9]	8 [6-9]	0.91[i]	0.03[k]
How much does pandemic COVID-19 affect you emotionally (e.g., makes you sad, angry, scared, and worried)? [median (IQR)]	7 [4-8]	7 [5-8]	0.93[i]	0.04[k]

Abbreviations: IQR, interquartile range; [a]N=9563; [b]N=9565; [c]N=9472; [d]N=9564; [e]N=6276; [f]N=6269; [g]N=6256; [h]N=6545; [i]Differences between cancer patients and free of cancer individuals were evaluated by the Kolmogorov-Smirnov test; [j]Differences between cancer patients and free of cancer individuals were evaluated by the chi2 test; [k]Effect size between cancer patients and free of cancer individuals was examined with Cohen's d; [l]Effect size between cancer patients and free of cancer individuals was examined with Cramér's V; Bold font indicates statistical significance ($p<0.05$).

Table S2. Male vs Female cancer participants comparisons in: socio-demographic characteristics, health behaviour data, COVID risk Self-Efficacy, Importance and Intentions, Coping (COPE B), Perceived Stress (PSS) data. Cognitive Affective, Mindfulness Scale data, Pro-Social Behaviour data, Brief Assessment of Family Functioning data, MSBS, Mental Health Continuum Short Form for Adults data, Positive and Negative Affect data, Illness (COVID) Perceptions data, Beliefs about COVID data.

		**Male**	**Female**	**p-value**	**Effect size**
Total number of participants		52	211		
**Socio-demographics characteristics**					
Age		55 [40, 64.5]	50 [40, 59]	**0.04**[l]	0.31[o]
Employment status [N (%)][a]	Working full time	28 (53.9)	106 (50.2)	**<.01**[m]	**0.24**[p]
	Working part time	5 (9.6)	43 (20.4)		
	Unemployed	1 (1.9)	23 (10.9)		
	On parental leave	0 (0.0)	6 (2.8)		
	Retired	18 (34.6)	33 (15.7)		
Working as A health professional [N (%)][b]	Yes	7 (14.3)	47 (23.0)	0.18[m]	0.08[p]
	No	42 (85.7)	157 (77.0)		
University student [N (%)][c]	Yes	3 (5.9)	23 (11.2)	0.26[m]	0.07[p]
	No	48 (94.1)	183 (88.8)		
Educational level [N (%)][a]	Primary education	0 (0.0)	2 (1.0)	0.66[m]	0.12[p]
	Secondary education	11 (22.0)	38 (18.2)		
	Higher education	39 (78.0)	169 (80.8)		
Marital status [N (%)][a]	Single	12 (23.5)	36 (17.2)	0.51[m]	0.13[p]
	In a relationship/Engaged/Married	36 (70.6)	141 (67.5)		
	Divorced/Widower	3 (5.9)	32 (15.3)		
Do you have children? [N (%)][a]	Yes	34 (65.4)	139 (65.9)	0.95[m]	0.00[p]
	No	18 (34.6)	72 (34.1)		
Living Situation [N (%)][a]	I live alone	8 (15.4)	39 (18.5)	0.68[m]	0.09[p]
	I live with my parents	5 (9.6)	14 (6.6)		
	I live with one of my parents	0 (0.0)	5 (2.4)		
	I live with my own family	38 (73.1)	146 (63.2)		

	I live with friends/roomates	1 (1.9)	7 (3.3)		
How big (approximately) is your inner living space? Please answer in squared meters. [median (IQR)]		102.5 (79.0, 150)	100 (67, 170)	0.72[n]	0.10[o]
How big (approximately) is your outer living space? Please answer in squared meters. [median (IQR)]		80 (8, 250)	26.4 (4, 200)	0.72[n]	0.13[o]
Total space in squared meters. [median (IQR)]		192 (100, 409)	180 (80, 360)	0.57[n]	0.13[o]
**Quarantine/self-isolation data**					
Weeks in quarantine or self-isolation [median (IQR)]		6 (5, 8)	5 (4, 7)	0.10[n]	0.13[o]
Since the quarantine began, have you needed to leave your house for work? [N (%)][d]	No	26 (50.0)	110 (52.4)	0.17[m]	0.14[p]
	Once only	1 (1.9)	13 (6.2)		
	A couple of times	9 (17.3)	48 (22.9)		
	More than 3 times per week	16 (30.8)	39 (18.5)		
Have your finances changed since being on quarantine? [N (%)][d]	Have got better	1 (1.9)	10 (4.8)	**0.02**[m]	0.17[p]
	Stayed the same	28 (53.9)	148 (70.5)		
	Have got worse	23 (44.2)	52 (24.7)		
Have you been able to obtain all the basic supplies you have tried to buy (e.g., soap, food items)? [N (%)][d]	Yes	49 (94.2)	198 (94.3)	0.99[m]	0.00[p]
	No	3 (5.8)	12 (5.7)		
**COVID Infection data**					
Have you been infected by COVID-19? [N (%)][a]	Yes	0 (0.0)	6 (2.8)	0.10[m]	0.13[p]
	No	50 (96.2)	180 (85.3)		
	I am not sure or have had symptoms but not diagnosed	2 (3.8)	25 (11.9)		
	Yes	0 (0.0)	3 (1.4)	0.68[m]	0.05[p]

Has your partner being infected by COVID-19? [N (%)][d]	No	49 (94.2)	194 (92.4)		
	I am not sure or (s)he has had symptoms but not diagnosed	3 (5.8)	13 (6.2)		
Has other than your partner being infected by COVID-19? [N (%)][a]	Yes	2 (3.9)	16 (7.5)	0.16[m]	0.12[p]
	No	49 (94.2)	178 (84.4)		
	I am not sure or (s)he has had symptoms but not diagnosed	1 (1.9)	17 (8.1)		
**Cancer-specific data**					
Are you currently on anticancer therapy? [N (%)][e]	Yes	11 (21.1)	48 (23.2)	0.75[m]	0.02[p]
	No	47 (78.9)	159 (76.8)		
If yes, your therapy is: [N (%)][f]	Adjuvant preventative	6 (24.0)	39 (32.5)	0.32[m]	0.12[p]
	For active disease	5 (20.0)	12 (10.0)		
	I did not receive therapy	14 (56.0)	69 (57.5)		
What scares you the most at this moment? [N (%)][g]	Cancer	16 (35.6)	70 (35.7)	0.98[m]	0.00[p]
	COVID	29 (64.4)	126 (64.3)		
What do you think can harm you more at this moment? [N (%)]	Cancer	11 (23.9)	61 (31.0)	0.35[m]	0.06[p]
	COVID	35 (76.1)	136 (69.0)		
What is your priority at this moment? [N (%)][h]	Cancer	12 (25.5)	40 (20.5)	0.45[m]	
	COVID	35 (74.5)	155 (79.5)		
**Health behavior**					
Do you smoke? [N (%)][a]	Yes	8 (15.4)	24 (11.4)	0.43[m]	0.05[p]
	No	44 (84.6)	187 (88.6)		
How many cigarettes did you smoke per day until the quarantine or self-isolation measures? [median (IQR)]		20 (15, 25)	12 (7, 20)	0.19[n]	0.09[p]

How many cigarettes did you smoke per day during the last week? [median (IQR)]		20 (15, 25)	12 (5, 18)	0.10[n]	0.10[p]
Difference of cigarettes before and current. [median (IQR)]		0 (-5, 0)	0 (-5, 0)	1.0[n]	0.03[p]
Do you consume alcohol? [N (%)][a]	Yes	24 (46.2)	85 (40.3)	0.44[m]	0.05[p]
	No	28 (53.8)	126 (59.7)		
How often did you consume alcohol per week until the quarantine or self-isolation measures? [median (IQR)]		2 (1, 4)	2 (1, 3)	0.89[n]	0.18[o]
How often did you consume alcohol per week before cups? [median (IQR)]		1 (1, 1)	1 (1, 1)	1.0[n]	-
How often did you consume alcohol during the last week? [median (IQR)]		2.5 (1, 5)	2 (1, 4)	0.93[n]	0.07[o]
How often did you consume alcohol before cups? [median (IQR)]		-	1 (1, 1)	-	-
Difference before and during. [median (IQR)]		0 (0, 0)	0 (0, 1)	0.56[n]	0.20[o]
Do you exercise? [N (%)][a]	Yes	38 (73.1)	137 (64.9)	0.27[m]	0.07[p]
	No	14 (26.9)	74 (35.1)		
How many times on average did you exercise per week until the quarantine or self-isolation measures? [N (%)][i]	Never	1 (2.6)	7 (5.1)	0.90[m]	0.06[p]
	Approximately 1-2 times	12 (31.6)	46 (33.6)		
	Approximately 3-4 times	17 (44.7)	59 (43.1)		
	At least 5-7 times	8 (21.1)	25 (18.2)		
	Never	1 (2.6)	5 (3.6)	0.41[m]	0.13[p]
	Approximately 1-2 times	9 (23.7)	48 (35.0)		

How many times on average did you exercise during the last week? [N (%)][i]	Approximately 3-4 times	20 (52.6)	52 (38.0)		
	At least 5-7 times	8 (21.1)	32 (23.4)		
On average how long did you exercise during the last week? [N (%)][i]	Not at all	0 (0.0)	5 (3.7)	0.61[m]	0.14[p]
	15 minutes or less	3 (7.9)	11 (8.0)		
	15-30 minutes	9 (21.0)	33 (24.1)		
	30-45 minutes	13 (34.2)	35 (25.5)		
	45 minutes - 1 hour	5 (13.2)	28 (20.4)		
	More than 1 hour	9 (23.7)	25 (18.3)		
Exercise current before [mean (SD)]		0.08 ± 0.71	0.06 ± 0.87	0.93[n]	0.02[p]
How would you rate your sleep quality overall? [N (%)][j]	Very good	11 (21.1)	34 (16.3)	0.69[m]	
	Fairly good	51 (40.4)	84 (40.2)		
	Neither good not bad	12 (23.1)	43 (20.6)		
	Fairly bad	6 (11.5)	41 (19.6)		
	Very bad	2 (3.9)	7 (3.3)		
How much has your sleep changed since quarantine? [N (%)][j]	I sleep more	9 (17.3)	41 (19.6)	0.91[m]	0.09[p]
	I sleep less	12 (23.1)	44 (21.1)		0.03[p]
	I sleep about the same	31 (59.6)	124 (59.3)		
What do you think your total screen time is during the last week (include TV, Laptop, computer, smartphone, tablet)? Please answer in hours per day. [median (IQR)]		5.5 (4, 8)	7 (5, 10)	**0.02**[n]	0.40[o]
What do you think your total screen time before (include TV, Laptop, computer, smartphone,		4 (2.25, 6.5)	4.5 (2, 8)	0.59[n]	0.10[o]

tablet)? Please answer in hours per day. [median (IQR)]				
Difference before and current. [median (IQR)]	1 (0, 3)	2 (0, 4)	0.31[n]	0.36[o]
**COVID risk factors data**				
Keeping distance from other people when going out. [median (IQR)]	10 (9, 10)	10 (9, 10)	1.0[n]	0.18[o]
Self-isolating, limiting unnecessary travelling according to national guidelines. [median (IQR)]	10 (9, 10)	10 (9, 10)	0.96[n]	0.05[o]
Washing hands regularly with water and soap. [median (IQR)]	10 (9, 10)	9 (9, 10)	0.11[n]	0.39[o]
I have the skills to get through this difficult situation. [median (IQR)]	7 (6, 7)	7 (6, 7)	0.95[n]	0.08[o]
I can deal with this difficult situation. [median (IQR)]	7 (6, 7)	7 (6, 7)	0.56[n]	0.08[o]
When facing difficulties in following the recommendations, I am certain that I will overcome them. [median (IQR)]	7 (6, 7)	7 (6, 7)	0.63[n]	0.23[o]
Compared to other people, I can follow these recommendations pretty well. [median (IQR)]	7 (6, 7)	7 (6, 7)	0.63[n]	0.44[o]
Even when things get tough, I can follow these recommendations quite well. [median (IQR)]	7 (6, 7)	6.5 (6, 7)	1.0[n]	0.16[o]
How important do you think it is to follow the recommendations to participate in social distancing, or	7 (6, 7)	7 (6, 7)	0.36[n]	0.15[o]

staying home as much as possible? [median (IQR)]				
How much do you intend on following the recommendations for social distancing (stay at home as much as possible) for the next week? [median (IQR)]	7 (6, 7)	7 (6, 7)	0.93[n]	0.30[o]
COVID risk self-efficacy [median (IQR)]	6.6 (5.8, 7)	6.6 (5.8, 7)	0.83[n]	0.16[o]
**Coping (COPE B)**				
COPEB self-distraction [median (IQR)]	6 (4, 7)	5 (4, 7)	1.0[n]	0.10[o]
COPEB active coping [median (IQR)]	6 (5, 7)	6 (5, 7)	0.88[n]	0.16[o]
COPEB denial [median (IQR)]	2 (2, 4)	2 (2, 3.5)	0.81[n]	0.05[o]
COPEB substance use [median (IQR)]	2 (2, 2)	2 (2, 3)	0.41[n]	0.36[o]
COPEB use of emotional support [mean (SD)]	4.56 ± 1.71	4.15 ± 1.67	0.06[l]	0.29[o]
COPEB use of instrumental support [median (IQR)]	4 (3, 5)	3.5 (2, 5)	0.17[n]	0.21[o]
COPEB behavior aldis engagement [median (IQR)]	2 (2, 3)	2 (2, 3.5)	1.0[n]	0.11[o]
COPEB venting [mean (SD)]	4.58 ± 1.63	4.40 ± 1.67	0.39[l]	0.13[o]
COPEB positive framing [median (IQR)]	6 (4, 7)	6 (5, 7)	1.0[n]	0.01[o]
COPEB planning [mean (SD)]	5.67 ± 1.65	5.67 ± 1.65	0.96[l]	0.01[o]
COPEB humour [mean (SD)]	4.51 ± 1.75	4.73 ± 1.85	0.32[l]	0.15[o]
COPEB acceptance [median (IQR)]	7 (6, 8)	8 (6, 8)	0.47[n]	0.09[o]
COPEB religion [median (IQR)]	4 (2, 6)	4 (2, 6)	0.55[n]	0.15[o]

COPEB self-blame [median (IQR)]		3 (2, 3)	3 (2, 4)	0.97[n]	0.02[o]
**Perceived Stress (PSS) data**					
PSS score [median (IQR)]		12 (9, 17)	15 (10, 21)	**0.03**[n]	0.29[o]
PSS level [N (%)][d]	Low	30 (57.7)	89 (42.4)	0.10[m]	0.13[p]
	Moderate	20 (38.5)	100 (47.6)		
	High	2 (3.8)	21 (10.0)		
**Cognitive Affective Mindfulness Scale data**					
CAMS mind flu qualities score [mean (SD)]		27.9 ± 3.70	27.2 ± 2.93	0.12[l]	0.24[o]
**Pro-Social Behaviour data**					
PSA pro socialness [mean (SD)]		22.9 ± 4.1	22.9 ± 4.0	0.87[l]	0.03[o]
**Social Support (OSS) data**					
OSS score [median (IQR)]		10.5 (8, 12)	10 (9, 12)	0.42[n]	0.16[o]
OSS level [N (%)][d]	Low	16 (30.8)	36 (17.1)	**0.03**[m]	0.16[p]
	Moderate	18 (34.6)	111 (52.9)		
	High	18 (34.6)	63 (30.0)		
**Brief Assessment of Family Functioning data**					
BAFFS score [median (IQR)]		5 (4, 6)	5 (4, 6)	1.0[n]	0.03[o]
**MSBS**					
MSBS reinforcement [median (IQR)]		3 (2.5, 3)	3 (3, 4)	1.0[n]	0.17[o]
MSBS boredom [median (IQR)]		2 (1.5, 2.5)	2 (1, 2.5)	0.97[n]	0.08[o]
MSBS lethargy [median (IQR)]		-0.53 (-1.59, 1.48)	-0.58 (-2.50, 1.25)	0.30[n]	0.13[o]
**Mental Health Continuum Short Form for Adults data**					
MHCSF score [median (IQR)]		45 (32, 54)	46 (37, 54)	0.54[n]	0.12[o]
MHCSF hedonic [median (IQR)]		11.5 (9, 13)	12 (9, 13)	0.55[n]	0.04[o]
MHCSF eudemonic social [mean (SD)]		12.2 ± 5.8	12.5 ± 5.8	0.75[n]	0.05[o]
MHCSF eudemonic psychological [median (IQR)]		22.5 (16, 25)	23 (18, 26)	0.62[n]	0.17[p]

MHCSF type [N (%)][k]	Languishing	3 (6.5)	16 (7.9)	0.44[m]	0.08[p]
	Moderately mental healthy	16 (34.8)	88 (43.8)		
	Flourishing	27 (58.7)	97 (48.3)		
**Positive and Negative Affect data**					
PANAS positive [median (IQR)]		31 (24.5, 37)	30 (25, 37)	0.89[n]	0.12[o]
PANAS negative [median (IQR)]		24 (19, 31.5)	26 (20, 33)	0.69[n]	0.11[o]
PANAS negative new [median (IQR)]		16 (12.5, 20.5)	17 (13, 23)	0.81[n]	0.09[o]
PANAS negative brief [median (IQR)]		8 (6, 10.5)	9 (7, 12)	0.44[n]	0.14[o]
**Illness (COVID) Perceptions data**					
How much COVID-19 does affects your life? [median (IQR)]		8 (6, 9)	8 (7, 9)	0.87[n]	0.07[o]
How long do you think COVID-19 will continue? [median (IQR)]		7 (6, 8)	7 (6, 8)	0.96[n]	0.06[o]
How much control do you feel you have over COVID-19? [median (IQR)]		7 (5, 8)	7 (5, 8)	0.99[n]	0.07[o]
How much do you think existing treatments help patients with COVID-19? [median (IQR)]		5 (3, 7)	6 (4, 7)	0.46[n]	0.29[o]
How much does COVID-19 worries you? [median (IQR)]		7.5 (5.5, 8)	7 (5, 9)	0.98[n]	0.07[o]
How well do you feel you understand what is happening with COVID-19? [median (IQR)]		7 (6, 9)	8 (7, 9)	0.08[n]	0.24[o]
How much does pandemic COVID-19 affect you emotionally (e.g., makes you sad, angry, scared, and worried)? [median (IQR)]		7 (4, 8)	7 (4, 8)	1.0[n]	0.04[o]
**Beliefs about COVID data**					

HBM susceptibility [mean (SD)]	11.3 ± 4.2	10.6 ± 4.1	0.27[n]	0.17[o]
HBM perceived severity [median (IQR)]	14 (10.5, 16.5)	14 (12, 17)	0.84[n]	0.17[o]
**Psychological Flexibility data**				
PsyFlex score [median (IQR)]	36 (30, 38)	35 (32, 38)	0.76[n]	0.07[o]

Abbreviations: SD, Standard Deviation; IIQR, interquartile range; [a]N=263; [b]N=253; [c]N=257; [d]N=262; [e]N=259; [f]N=145; [g]N=241; [h]N=243; [i]N=175; [j]N=261; [k]N=247;

[l]Differences between males and females were evaluated by the t- test.

[m]Differences between males and females were evaluated by the chi-square test.

[n]Differences between males and females were evaluated by the Kolmogorov-Smirnov test.

[o]Effect size between males and females was examined with Cohen's d.

[p]Effect size between males and females was examined with Cramér's V.

Bold font indicates statistical significance ($p<0.05$).

Table S3. Socio-demographic characteristics, health behavior data, COVID risk Self-Efficacy, Importance and Intentions, Coping (COPE B), Perceived Stress (PSS) data. Cognitive Affective, Mindfulness Scale data, Pro-Social Behaviour data, Brief Assessment of Family Functioning data, MSBS, Mental Health Continuum Short Form for Adults data, Positive and Negative Affect data, Illness (COVID) Perceptions data, Beliefs about COVID data, separately for cancer patients with cancer priority and for COVID priority.

		**Cancer priority**	**COVID priority**	**p-value**	**Effect size**
**Total number of the participants [N]**		52	191		
**Socio-demographics characteristics**					
Median inner living space [square meters (IQR)]		100 [71, 180]	100 [68, 160]	0.84[g]	0.05[i]
Median outer living space [square meters (IQR)]		57.5 [9, 185]	27.9 [4, 250]	0.70[g]	0.15[i]
Median total living space [square meters (IQR)]		201 [98, 310]	180 [81, 450]	0.68[g]	0.15[i]
**Quarantine/self-isolation data**					
Weeks in quarantine or self-isolation		6 [4, 7]	65[4, 7]	0.99[g]	0.15[i]
Since the quarantine began, have you needed to leave your house for work? [N (%)]	No	23 (44.2)	102 (53.7)	0.44[f]	0.11[j]
	Once only	2 (3.9)	11 (5.8)		
	A couple of times	16 (30.8)	40 (21.1)		

	More than 3 times per week	11 (21.2)	37 (19.5)		
Have your finances changed since being on quarantine? [N (%)][a]	Have got better	0 (0.0)	9 (4.7)	0.22[f]	0.11[j]
	Stayed the same	34 (65.4)	127 (66.8)		
	Have got worse	18 (34.6)	54 (28.4)		
Have you been able to obtain all the basic supplies you have tried to buy (e.g., soap, food items)? [N (%)][a]	Yes	50 (96.2)	178 (93.7)	0.50[f]	0.04[j]
	No	2 (3.9)	12 (6.3)		
**COVID infection data**					
Have you been infected by COVID-19? [N (%)][b]	Yes	0 (0.0)	6 (3.1)	0.17[f]	0.12[j]
	No	49 (94.2)	162 (84.8)		
	I am not sure or have had symptoms but not diagnosed	3 (5.8)	23 (12.0)		
Has your partner being infected by COVID-19? [N (%)][a]	Yes	0 (0.0)	4 (2.1)	0.41[f]	0.08[j]
	No	50 (96.2)	173 (91.1)		
	I am not sure or have had symptoms but not diagnosed	2 (3.8)	13 (6.8)		
Has your partner being infected by COVID-19? [N (%)][b]	Yes	5 (9.6)	14 (7.3)	0.83[f]	0.05[j]
	No	43 (82.7)	164 (85.9)		
	I am not sure or have had symptoms but not diagnosed	4 (7.7)	13 (6.8)		
**Health Behavior**					
Do you smoke? [N (%)][b]	Yes	5 (9.6)	23 (12.0)	0.63[f]	0.03[j]
	No	47 (90.4)	168 (88.0)		
How many cigarettes did you smoke per day until the quarantine or self-isolation measures? [median (IQR)]		13.5 [5-30]	13 [8-20]	0.95[g]	0.08[i]
How many cigarettes did you smoke per day during the last week? [median (IQR)]		20 [20-20]	12 [10-20]	0.18[g]	0.12[i]
Difference of cigarettes before and current. [median (IQR)]		0 [-10-36.5]	0 [0-0]	1.0[g]	0.20[i]
Do you consume alcohol? [N (%)][b]	Yes	17 (32.7)	86 (45.0)	0.11[f]	0.10[j]
	No	35 (67.3)	105 (56.0)		
How often did you consume alcohol per week until the quarantine or self-isolation measures? [median (IQR)]		2 [2-3]	2 [1-3]	0.63[g]	0.03[i]

How often did you consume alcohol per week before cups? [median (IQR)]		-	1 [1-1]	-	-
How often did you consume alcohol during the last week? [median (IQR)]		3 [1-4]	2 [1-4]	0.77[g]	0.06[i]
How often did you consume alcohol before cups? [median (IQR)]		-	1 [1-1]	-	-
Difference before and during. [median (IQR)]		0 [0-1]	0 [0-1]	1.0[g]	0.18[i]
Do you exercise? [N (%)][b]	Yes	37 (71.2)	123 (64.4)	0.36[f]	0.06[j]
	No	15 (28.9)	68 (35.6)		
How many times on average did you exercise per week until the quarantine or self-isolation measures? [N (%)][c]	Never	2 (5.4)	6 (4.9)	0.50[f]	0.12[j]
	Approximately 1-2 times	16 (43.2)	40 (32.5)		
	Approximately 3-4 times	15 (40.5)	52 (42.3)		
	At least 5-7 times	4 (10.8)	25 (20.3)		
How many times on average did you exercise during the last week? [N (%)][d]	Never	0 (0.0)	6 (4.9)	0.13[f]	0.19[j]
	Approximately 1-2 times	17 (46.0)	38 (30.9)		
	Approximately 3-4 times	16 (43.2)	51 (41.5)		
	At least 5-7 times	4 (10.8)	28 (22.8)		
On average how long did you exercise during the last week? [N (%)][d]	Not at all	0 (0.0)	5 (4.1)	0.66[f]	0.14[j]
	15 minutes or less	4 (10.8)	9 (7.3)		
	15-30 minutes	11 (29.7)	26 (21.1)		
	30-45 minutes	9 (24.3)	36 (29.3)		
	45 minutes - 1 hour	7 (18.9)	23 (18.7)		
	More than 1 hour	6 (16.2)	24 (19.5)		
Exercise current before [mean (SD)]		0.08 ± 0.92	0.04 ± 0.82	0.92[h]	0.05[i]
How would you rate your sleep quality overall? [N (%)][e]	Very good	9 (17.3)	32 (16.9)	0.44[f]	0.12[j]
	Fairly good	16 (30.8)	77 (40.7)		
	Neither good not bad	16 (30.8)	38 (20.1)		
	Fairly bad	10 (19.2)	34 (18.0)		
	Very bad	1 (1.9)	8 (4.2)		
How much has your sleep changed since quarantine? [N (%)][e]	I sleep more	12 (23.1)	34 (18.0)	0.35[f]	0.10[j]
	I sleep less	8 (15.4)	46 (24.3)		
	I sleep about the same	32 (61.5)	109 (57.7)		
What do you think your total screen time is during the last week (include TV, Laptop, computer, smartphone, tablet)? [median (IQR)]		6 [4-8]	6 [4-10]	0.73[g]	0.08[i]

What do you think your total screen time before (include TV, Laptop, computer, smartphone, tablet)? [median (IQR)]	4 [2-6.5]	4 [2-8]	0.76[g]	0.19[i]
Difference before and current. [median (IQR)]	2 [0-3.25]	2 [0-4]	0.98[g]	0.08[i]
**COVID risk Self-Efficacy, Importance and Intentions**				
I have the skills to get through this difficult situation. [median (IQR)]	7 [6-7]	7 [6-7]	0.94[g]	0.28[i]
I can deal with this difficult situation. [median (IQR)]	7 [6-7]	7 [6-7]	0.99[g]	0.38[i]
When facing difficulties in following the recommendations, I am certain that I will overcome them. [median (IQR)]	6 [6-7]	7 [6-7]	0.85[g]	0.19[i]
Compared to other people, I can follow these recommendations pretty well. [median (IQR)]	7 [6-7]	7 [6-7]	0.66[g]	0.04[i]
Even when things get tough, I can follow these recommendations quite well. [median (IQR)]	6.5 [6-7]	7 [6-7]	1.0[g]	0.07[i]
COVID risk self-efficacy [median (IQR)]	6.6 [6-7]	6.6 [5.8-7]	0.99[g]	0.22[i]
How important do you think it is to follow the recommendations to participate in social distancing, or staying home as much as possible? [median (IQR)]	7 [5-7]	7 [7-7]	**<.01**[g]	0.27[i]
How much do you intend on following the recommendations for social distancing (stay at home as much as possible) for the next week? [median (IQR)]	7 [6-7]	7 [6-7]	0.50[g]	0.21[i]
**Coping (COPE B)**				
COPEB self-distraction [median (IQR)]	6 [5-7]	6 [4-7]	1.0[g]	0.02[i]
COPEB active coping [median (IQR)]	6 [5-8]	6 [5-7]	0.66[g]	0.23[i]
COPEB denial [median (IQR)]	3 [2-4.5]	2 [2-4]	0.55[g]	0.31[i]
COPEB substance use [median (IQR)]	2 [2-2]	2 [2-2]	1.0[g]	0.07[i]
COPEB use of emotional support (mean (SD))	4.59 ± 1.54	4.58 ± 1.75	1.0[g]	0.02[i]
COPEB use of instrumental support [median (IQR)]	4 [3.5-5]	4 [3-5]	0.69[g]	0.11[i]
COPEB behavior aldis engagement [median (IQR)]	2 [2-3]	2 [2-3]	1.0[g]	0.05[i]
COPEB venting (mean (SD))	5.02 ± 1.80	4.52 ± 1.55	0.15[g]	0.31[i]
COPEB positive framing [median (IQR)]	6.5 [5-8]	6 [4-7]	0.44[g]	0.28[i]
COPEB planning (mean (SD))	6.00 ± 1.66	5.63 ± 1.57	0.29[g]	0.22[i]
COPEB humour (mean (SD))	4.90 ± 1.61	4.36 ± 1.77	0.13[g]	0.31[i]

COPEB acceptance [median (IQR)]		7 [6-8]	7 [6-8]	1.0[g]	0.01[i]
COPEB religion [median (IQR)]		4 [2-6]	3 [2-6]	0.77[g]	0.14[i]
COPEB self-blame [median (IQR)]		3 [2-4]	3 [2-4]	1.0[g]	0.06[i]
**Perceived Stress (PSS) data**					
PSS score [median (IQR)]		14.5 [11-19]	15 [10-21]	0.93[g]	0.06[i]
**Cognitive Affective Mindfulness Scale data**					
CAMS mind flu qualities score (mean (SD))		27.5 ± 2.77	27.2 ± 3.07	0.79[g]	0.09[i]
**Pro-Social Behaviour data**					
PSA pro socialness score (mean (SD))		23.1 ± 3.7	22.8 ± 4.2	0.33[g]	0.10[i]
**Brief Assessment of Family Functioning data**					
BAFFS score [median (IQR)]		5 [4-6]	6 [4-6]	1.0[g]	0.10[i]
**MSBS**					
MSBS reinforcement [median (IQR)]		3 [3-3.5]	3 [3-3]	1.0[g]	0.12[i]
MSBS boredom [median (IQR)]		1.5 [1-2.5]	2 [1.5-2.5]	0.34[g]	0.23[i]
**Mental Health Continuum Short Form for Adults data**					
MHCSF score [median (IQR)]		46 [38.5-52]	46 [34.5-54]	0.70[g]	0.09[i]
MHCSF hedonic [median (IQR)]		11 [9-13]	12 [9-13]	1.0[g]	0.01[i]
MHCSF eudemonic social (mean (SD))		12.4 ± 5.3	12.6 ± 5.9	0.94[g]	0.03[i]
MHCSF eudemonic psychological [median (IQR)]		23 [19-27]	22 [17-25]	0.44[g]	0.28[i]
MHCSF type	Languishing	1 (2.1)	17 (9.4)	0.26[g]	0.10[j]
	Moderately mental healthy	21 (44.7)	75 (41.7)		
	Flourishing	25 (53.2)	88 (48.9)		
**Positive and Negative Affect data**					
PANAS positive [median (IQR)]		32 [28.5-38]	30 [24-37]	**0.03**[g]	0.34[i]
PANAS negative [median (IQR)]		24 [20-33]	26 [21-35]	0.44[g]	0.19[i]
**Illness (COVID) Perceptions data**					
How much COVID-19 does affects your life? [median (IQR)]		8 [6-9]	8 [7-9]	0.60[g]	0.16[i]
How long do you think COVID-19 will continue? [median (IQR)]		7 [5-8]	7 [6-8]	0.97[g]	0.04[i]
How much control do you feel you have over COVID-19? [median (IQR)]		7 [5-8]	7 [5-8]	0.92[g]	0.08[i]
How much do you think existing treatments help patients with COVID-19? [median (IQR)]		5.5 [3.5-8]	6 [4-7]	1.0[g]	0.02[i]

How much does COVID-19 worries you? [median (IQR)]	6 [4-8]	8 [6-9]	**<.01**[g]	0.65[i]
How well do you feel you understand what is happening with COVID-19 [median (IQR)]	8 [6-9]	8 [6-9]	0.86[g]	0.08[i]
How much does pandemic COVID-19 affect you emotionally (e.g., makes you sad, angry, scared, and worried)? [median (IQR)]	6 [4-8]	7 [5-8]	0.11[g]	0.34[i]
**Beliefs about COVID data**				
HBM susceptibility (mean (SD))	10.0 ± 4.3	11.1 ± 4.0	0.34[g]	0.29[i]
HBM perceived severity [median (IQR)]	12.5 [9-15]	15 [13-17]	**<.01**[g]	0.76[i]

Abbreviations: SD, Standard Deviation; IQR, interquartile range; [a]N=242; [b]N=243; [c]N=150; [d]N=160; [e]N=241;

[f] Differences between cancer priority and COVID priority were evaluated by the chi-square test.

[g] Differences between cancer priority and COVID priority were evaluated by the Kolmogorov-Smirnov test.

[h] Differences between cancer priority and COVID priority were evaluated by the t- test.

[i]Effect size between cancer priority and COVID priority was examined with Cohen's d.

[j]Effect size between cancer priority and COVID priority was examined with Cramér's V.

Bold font indicates statistical significance (p<0.05).
